# Plant Immunity Is Compartmentalized and Specialized in Roots

**DOI:** 10.3389/fpls.2018.01692

**Published:** 2018-11-28

**Authors:** Coralie Chuberre, Barbara Plancot, Azeddine Driouich, John P. Moore, Muriel Bardor, Bruno Gügi, Maïté Vicré

**Affiliations:** ^1^Normandie Univ, UNIROUEN, Laboratoire Glycobiologie et Matrice Extracellulaire Végétale EA4358, Rouen, France; ^2^Fédération de Recherche “NORVEGE”- FED 4277, Rouen, France; ^3^Department of Viticulture and Oenology, Faculty of AgriSciences, Institute for Wine Biotechnology, Stellenbosch University, Matieland, South Africa; ^4^Institut Universitaire de France, Paris, France

**Keywords:** elicitor, systemic acquired resistance, root immunity, root border cells, environment

## Abstract

Roots are important organs for plant survival. In recent years, clear differences between roots and shoots in their respective plant defense strategies have been highlighted. Some putative gene markers of defense responses usually used in leaves are less relevant in roots and are sometimes not even expressed. Immune responses in roots appear to be tissue-specific suggesting a compartmentalization of defense mechanisms in root systems. Furthermore, roots are able to activate specific defense mechanisms in response to various elicitors including Molecular/Pathogen Associated Molecular Patterns, (MAMPs/PAMPs), signal compounds (e.g., hormones) and plant defense activator (e.g., β-aminobutyric acid, BABA). This review discusses recent findings in root defense mechanisms and illustrates the necessity to discover new root specific biomarkers. The development of new strategies to control root disease and improve crop quality will also be reviewed.

## Introduction

In the natural environment, plants are continuously exposed to diverse pathogens that affect crop production and food security. In the last decade, considerable progress has been made to understand the molecular and cellular interactions between pathogens and plants (Lai and Mengiste, 2013; Zhang et al., 2013). The cell wall represents the first line of plant defense acting as a preformed barrier against pathogen invasion. Activation of inducible defense reactions is based on the plant’s ability to detect the presence of microorganisms through the recognition of highly conserved molecular patterns called MAMPs (Microbe-Associated Molecular Patterns). These conserved patterns are foreign as they derive directly from either non-pathogenic microorganisms (Henry et al., 2012) or from pathogenic microorganisms, e.g., bacterial flagella (Chinchilla, 2006), peptidoglycan (Gust et al., 2007), fungal chitin (Zipfel and Felix, 2005). These pathogenic derived patterns are commonly referred to as PAMPs (Pathogen Associated Molecular Patterns). Plants also recognize endogenous signals released by the plant itself, under pathogen pressure (Klarzynski and Fritig, 2001) or during abiotic stress (Choi and Klessig, 2016). These biotic and abiotic stress related patterns are called DAMPs (Damage-Associated Molecular Patterns) (Boller and Felix, 2009). All of these molecular patterns, also known as general elicitors (Henry et al., 2012), are recognized by pattern recognition receptors (PRRs) present at the cell surface (Newman et al., 2013; De Lorenzo et al., 2018). In this review, the generic term elicitor refers to any compound that triggers plant responses in various ways: MAMPs/PAMPs/DAMPs, signaling compounds (salicylic acid, methyl-jasmonate), priming agents and plant defense activators (PDAs) used for agricultural practices (e.g., β-aminobutyric acid, BABA). The perception of such elicitors triggers the activation of a non-host resistance known as Pathogen- or Pattern-Triggered Immunity (PTI) (Jones and Dangl, 2006; Bigeard et al., 2015). PTI contributes to host defense against a broad range of pathogens. The current knowledge around plant immunity is predominantly focused on the aerial part of the plant. However, the root is the organ that encounters the highest microorganism density and diversity being within the rhizosphere (Torsvik and Ovreas, 2002; Jacobs et al., 2011). In this review, we aim to provide new insights regarding root immune system responses to PAMPs. Furthermore, the ability of pathogen mimicking molecules, referred as PDAs on stimulating root defense mechanisms will be discussed.

## The Root System as a Major Site for Microbe Entry

The rhizosphere is highly enriched in microorganisms with up to 10^6^–10^9^ bacteria, 10^5^–10^6^ fungi and 10^1^–10^2^ nematodes per gram of soil (Watt et al., 2006; Mendes et al., 2013). These organisms can be either beneficial, such as plant growth-promoting rhizobacteria (PGPR) (Beneduzi et al., 2012), or pathogenic, such as *Fusarium* spp. Soil-borne such as *Fusarium, Pythium*, or *Phytophthora* infect the roots of a variety of crops resulting in significant economic losses. Thus, roots represent an important opportunistic entryway for a number of soil pathogens. Pathogens can penetrate roots through natural apertures present at the junction between the main and lateral roots such as epidermal cracks (Perrine-Walker et al., 2007) or through young growing tissues which lack secondary cell walls (Okubara and Paulitz, 2005). Amongst pathogenic organisms, vascular pathogens penetrate the root system to infect and cause symptoms within the aerial parts of host plants such as their leaves. For example, the fungus *Colletotrichum graminicola*, responsible of maize anthracnose is able to infect roots even though it is generally not considered to be a root disease pathogen (Sukno et al., 2008). In rice, the fungus *Magnaporthe grisea* (Sesma and Osbourn, 2004), typically thought to be a foliar pathogen, was able to spread from infected roots to leaves. *Fusarium oxysporum* f. sp. *vasinfectum* and *Verticillium longisporum* infect root vascular tissue (Dowd et al., 2004) and moves upwards to the foliage (Iven et al., 2012). It is therefore essential for roots to detect soil pathogens and initiate defense responses to limit pathogen infection and spread. Although root infections have a negative impact on crop production (Okubara and Paulitz, 2005), most plant defense studies are focused on leaves (Balmer and Mauch-Mani, 2013) rather than on roots. To date, only few investigations are dedicated to root defenses as root systems are more complex to study because of their general inaccessibility. In addition, soil-borne microorganisms are not easily culturable *in vitro*. Less than 1% of soil microorganisms are currently characterized and culturable under laboratory conditions (Singh et al., 2004).

## Pamps Recognition in Roots

Recent studies have highlighted that roots are able to perceive the presence of pathogens and induce the PTI response. Induction of defense mechanisms is shown to occur in response to a wide range of elicitors including the flagellin-derived peptide elicitor Flagellin22 (Flg22), fusaric acid, peptidoglycan (PGN), Methyl-jasmonate (MeJA) and extracts from *Pectobacterium atrosepticum* (Millet et al., 2010; Plancot et al., 2013; Gotté et al., 2015, 2016; Koroney et al., 2016). Treatment with Flg22 was shown to enhance resistance to microbial invasion in roots by inducing reactive oxygen species (ROS) accumulation, callose deposition and the production of antimicrobial compounds during PTI response (Millet et al., 2010; Tran T.M. et al., 2016). These defense responses clearly show that root cells are able to perceive elicitors, suggesting the presence of cell-surface receptors.

To confirm the existence of MAMPs/PAMPs receptors, the root responses were investigated in an Arabidopsis *fls2* mutant lacking a functional Flg22 receptor. The PTI response to Flg22 was completely abolished in the *fls2* mutant confirming the existence of specific PRRs in Arabidopsis roots (Gómez-Gómez and Boller, 2000; Gómez-Gómez, 2004; Robatzek et al., 2006; Millet et al., 2010). It has been shown that all Arabidopsis root tissues, including the root-cap derived border-like cells (BLCs), have the capacity to activate immune responses (Plancot et al., 2013; Wyrsch et al., 2015). This suggests that Flg22 receptor (FLS2) is present in all root tissues. Further studies demonstrated significant differences in the expression of the gene *FLS2* in various root tissues (Beck et al., 2014). Indeed, intensity of the responses observed was more pronounced within the inner root tissues believed to be due to enriched receptor distribution and density in the endodermis and stele cells (Beck et al., 2014; Wyrsch et al., 2015). However, even if the *FLS2* promoter activity is mainly present in the root stele, the expression of *FLS2* expanded to cortical and epidermal cell layers when roots are grown under non-sterile or biotic stress conditions (Beck et al., 2014; Wyrsch et al., 2015). Moreover, *FLS2* expression is also shown to be regulated in a developmental-dependent manner (Beck et al., 2014). For example, the *FLS2* promoter activity is highly expressed in primordia and in growing lateral roots. However when these lateral roots reach a mature developmental stage, the *FLS2* expression became restricted to the vascular cylinder of the developing lateral roots (Beck et al., 2014). Therefore, these findings suggest that the expression of MAMPs receptors are limited and restricted to the most vulnerable sites for pathogen entryway. Activation of PTI was therefore induced only when PAMPs perception recognizes a significant specific threat to the plant itself. This is especially true when MAMPs/PAMPs are detected in the pericycle or in the vascular system. However, PAMPs perception by roots was also reported to differ depending on the elicitor type (e.g., fusaric acid, chitin or mycelium extract from *Fusarium oxysporum*) (Millet et al., 2010; Plancot et al., 2013) and according to the specific plant species under study (Tran T. et al., 2016).

In the rhizosphere, roots are constantly exposed to MAMPs. Therefore, roots need to distinguish between microbial “friend or foe,” i.e., recognizing beneficial microbes from pathogenic microbes. It is thus necessary that the root system modulates the defense responses that are associated with their recognition in order to avoid a constant and energetically expensive activation of the PTI. Consequently, beneficial microbes have to escape recognition by the root receptors in order to establish root interactions. One hypothesis is that *FLS2* expression in roots can be sufficiently low to allow beneficial bacteria to colonize root tissue without triggering PTI defense responses (Millet et al., 2010). Both beneficial and pathogen micro-organisms are able to modulate root immune responses suggesting a fine-tuned molecular dialog. Such interaction is complex because it involves the interactions with beneficial microbes, which the plant must encourage without threatening its own survival. Interactions between microbiota and roots were recently well reviewed in Hacquard et al., 2017. For more details, the readers are invited to refer to this paper.

## PTI Responses in Roots

**FIGURE 1:**
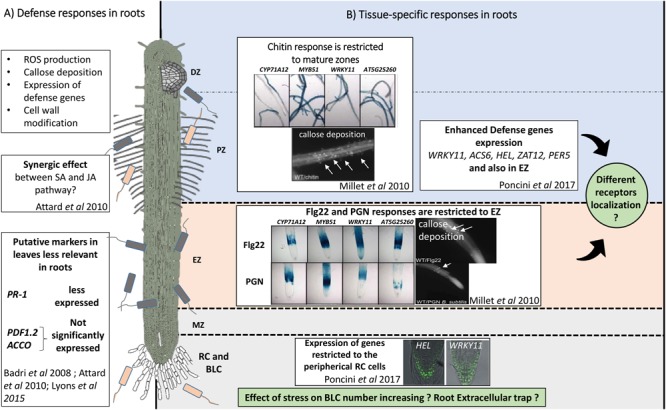
Overview of global and tissue-specific defense responses in *Arabidopsis thaliana* root. **(A)** Early PAMPs-triggered immunity (PTI) are common to leaves and roots. Production of ROS, callose deposition, an increase of defense genes leading to cell wall modification have been described in leaves and roots tissues. However, differences are noticed: the antagonistic effect of Salicylic acid (SA) and Jasmonic acid (JA) is not observed in *A. thaliana* root in response to *Phytophthora parasitica* infection (Attard et al., 2010). Markers of SA (PR-1), JA (PDF1.2) and ET (ACCO) expressed in leaves are also less relevant in roots (Badri et al., 2008; Attard et al., 2010; Lyons et al., 2015). **(B)** Root responses show tissue-specificity. In this schematic representation of a root, different zones can be distinguished: DZ, differentiation zone; PZ, root hair zone; EZ, elongation zone; MZ, meristematic zone; RC and BLCs, root cap and border-like cells. Millet et al. (2010), used various defense gene promotors in fusion to GUS in order to evaluate the defense gene expression in the different zone of the root after elicitation with various MAMPs. In response to chitin elicitation, gene expression occurred throughout the entire mature zones (DZ and PZ). In contrast, with Flg22 and PGN, the response is restricted to EZ. In this work, callose deposition was also studied. Furthermore, in root caps, it has been shown that in response to Flg22, expression of defense genes such as WRKY11 and HEL was restricted to the peripheral RC cells (Poncini et al., 2017). This compartmentalized response might suggest tissue-specific elicitor receptors localization. SA, Salicylic acid; JA, Jasmonic acid; ET, Ethylene; PR-1, Pathogenesis related proteins 1; PDF1.2, Plant defensin family 1.2; ACCO, Amino-cyclopropane-carboxylate oxidase myrosinase binding protein; CYP71A12, Cytochrome P450 family 71 polypeptide; MYB51, Transcription factor for the regulation of indole-glucosinolate biosynthesis; WRKY11, Negative regulator of basal resistance; AT5G25260, Nodulin-like protein of unknown function; ACS6 : 1-aminocyclopropane-1-carboxylate synthase 6; HEL, Hevein-like protein; ZAT12, Zinc-finger protein; PER5, Peroxidase superfamily protein; Flg22, Flagellin fragment of 22 amino-acid; PGN, Peptidoglycan. 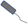: Pathogen microorganisms 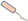: Beneficial microorganisms. Copyright by the American Society of Plant Biologists.

Previous studies performed in roots indicated that PTI includes molecular events such as the production of ROS (Poncini et al., 2017), transcriptional reprogramming (Boller and Felix, 2009; Millet et al., 2010), callose deposition (Millet et al., 2010) and modified extensin distribution within the cell wall (Ribeiro, 2006; Wojtasik et al., 2011; Plancot et al., 2013; **Figure 1A**). Phytohormones including salicylic acid (SA), jasmonic acid (JA), and ethylene (ET), are also implicated in root defense against pathogens (Tytgat et al., 2013; Papadopoulou et al., 2018). However, the antagonistic interactions of the two hormones JA and SA reported in leaves did not always follow the infection process in, for example, the roots of *Arabidopsis thaliana* (Badri et al., 2008; Attard et al., 2010). In addition, the regulation of defense genes by hormonal elicitation was reported to be organ-specific and differed both quantitatively and qualitatively between aerial and below ground organs in the non-model plants *Brassica oleracera* and *Brassica rapa* (Tytgat et al., 2013; Papadopoulou et al., 2018). Differences regarding the diversity and levels of antimicrobial compound produced occur between roots and leaves suggesting that different regulation mechanisms exist for defense in roots. For example in rice, the expression of typical defense-related genes such as pathogenesis related-proteins (PR-1, PR-10) are rapidly, but transiently, transcribed during the early stages of root infection whereas in leaves the same transcripts continue to accumulate to high levels during later stages of infection (Marcel et al., 2010). Consequently, PR-1 which is a characteristic salicylic acid (SA) marker highly expressed in Arabidopsis leaves, is found to be produced at very low concentrations in maize (Balmer et al., 2013) and rice (Marcel et al., 2010) in response to pathogen attack. In *B. rapa*, PR-1 shows also differential regulation in response to hormonal elicitation in both organs (Papadopoulou et al., 2018) confirming that shoots and roots can adopt specific defense responses (Tytgat et al., 2013). Other important proteins controlling plant responses to pathogen are found in leaves such as JA markers (plant defensin: PDF1.2), ET markers (amino-cyclopropane-carboxylate oxidase myrosinase binding protein: ACCO), glucosinolate markers (enzyme involves in glucosinolate biosynthesis: CYP79B2). However, these same biomarkers are not significantly induced in Arabidopsis roots (Badri et al., 2008; Attard et al., 2010; Lyons et al., 2015) suggesting they are leaf specific. Taking glucosinolate biosynthesis marker genes as an example we consider their differential expression in roots versus leaves. Glucosinolates are major nitrogen and sulfur containing secondary metabolites that alleviate biotic and abiotic stress in plant species belonging to Brassicaceae (Martínez-Ballesta et al., 2013; Augustine and Bisht, 2015). As compared to leaves, JA induces glucosinolate biosynthesis genes very weakly in roots as glucosinolates are found to be constitutively present in the below ground organs (i.e., roots) of *B. oleracera* (Tytgat et al., 2013). It is suggested that the constitutive high levels of glucosinolates in roots is an efficient approach to counteract rhizosphere pathogen attack versus an inducible synthesis approach. Similarly, the Endoplasmic Reticulum (ER)-bodies are reported to be constitutively produced in roots of *Arabidopsis thaliana* whereas their formation in leaves is only found upon wounding or MeJA treatment (Matsushima, 2002). ER-bodies are organized into network structures that accumulate defense proteins such as β-glucosidase (Nakano et al., 2014). In stress-free conditions, ER-bodies are reported to occur in roots (Nakano et al., 2014, 2017). However, these root ER-bodies are not uniformly formed and have been reported to be absent in some tissues including the quiescent zone, the endodermis and the stele (Gotté et al., 2015). Interestingly, exogenous application of JA or MeJA in roots were shown to have an effect on both the morphology and enzymatic activity of the ER-bodies (Gotté et al., 2015). Such data suggest that ER-bodies are part of the PTI response in roots. Based on these studies, there is a need to carefully select specific gene markers, both according to the plant species and the organs (e.g., aerial or belowground organs) under investigation.

## Tissue-Specific Responses in Roots

Many pathogens are reported to infect their hosts through specific regions of the root termed root zones (RZs) (Gunawardena and Hawes, 2002; Oren et al., 2003; Cannesan et al., 2011). This suggests that root tissues differ in their susceptibility to pathogens and therefore their inherent resistance capacities. For example, spatiotemporal events in tomato roots are important to limit *Ralstonia solanacearum* from spreading and infecting the entire plant (Caldwell et al., 2017). In pea, *Aphanomyces euteiches* infection is restricted in roots suggesting the existence of tissue specific responses (Cannesan et al., 2011). The differential sensitivities found in root tissues can be partially explained by the spatial localization of pisatin, a phytoalexin that accumulates in response to pathogen infection. It was shown that pisatin accumulates mainly in RZs that escaped pathogen infection [e.g., root cap cells (RC) and differentiation zone (DZ)]. Pisatin concentration is however significantly lower in the more susceptible elongation zone (EZ) (Cannesan et al., 2011). The greater susceptibility of the root EZ is explained by the yougth of elongating cells which have not fully developed protective tissue such as their epidermis barrier (Wyrsch et al., 2015). Thus, innate immunity responses appeared to be enhanced in EZs as compared to the other parts of the root system (Millet et al., 2010; Poncini et al., 2017). In Arabidopsis roots, Flg22 or PGN are elicitors derived from bacteria that trigger callose deposition in the EZ (Millet et al., 2010; **Figure 1B**). The expression of defense genes such as the cytochrome P450 *CYP71A12* and the transcription factor MYB51, that are involved in the biosynthesis compound toxic to pathogens, are activated after Flg22 treatment in EZs (Millet et al., 2010). These genes are also expressed in response to root-knot nematodes in EZs (Teixeira et al., 2016). In contrast, in response to chitin, an elicitor derived from fungal cell walls, *CYP71A12, MYB51* expression and callose deposition was observed in all mature parts of the RZ (Millet et al., 2010). In addition, early MAMP-signaling marker WRKY11 and the ROS markers ZAT12 and PER5 were also reported to be mainly induced in the EZ and DZ (Poncini et al., 2017). Indeed, YFP marker fusion constructs expressed in the EZ were only visible in the epidermal layer without elicitor treatment and expressed in the central tissues few hours after elicitor treatment (e.g., pMYB51::YFP_N_ and pZAT12::YFP_N_). Whereas in the root tip, YFP fluorescence was restricted to the peripherical root cap cells (e.g., pWRKY11::YFP_N_ and pHEL::YFP_N_) (Poncini et al., 2017). These data suggest that PTI induction can be restricted to specific root tissue zones that are critical for successful infection by invading pathogen (Millet et al., 2010; Teixeira et al., 2016).

Newly generated tissues are preferential zones of infection by soil pathogens. However, most of the nascent root tips remain uninfected even if other root tissues develop lesions (Wen et al., 2006). Indeed, the root tip inoculated with *Nectria haematococca* remained devoid of infection whereas the EZ was almost entirely infected. (Gunawardena and Hawes, 2002). This root tip tissue-specific resistance is correlated spatially with the presence of border cells (BCs) surrounding the root cap periphery (Driouich et al., 2013; Koroney et al., 2016). These BCs, which are individually separated from the root tip immediately after water contact (Hawes et al., 2000) are embedded in a thick mucilage and respond to both biotic (Driouich et al., 2013) and abiotic stresses (Miyasaka and Hawes, 2001; Cai et al., 2011). Several studies have demonstrated that BCs act as a physical and chemical barrier to fungi (Gunawardena and Hawes, 2002; Cannesan et al., 2011), bacteria (Hawes and Pueppke, 1987), zoospores (Goldberg et al., 1989), and nematodes infection (Zhao et al., 2000). These studies suggest a special role for the root cap and associated tissues in plant defense (Hawes et al., 2016; Tran T.M. et al., 2016). Therefore, it was suggested that BCs act as “sentries” providing specialized protection of the root cap and root meristems (Plancot et al., 2013). Root BCs were shown to attract pathogens such as oomycetes and nematodes (*via* chemotaxis) in order to subsequently neutralize them using antimicrobial mucilage traps (Zhao et al., 2000; Hawes et al., 2016). It is reported that root BCs attract zoospores (Zhao et al., 2000) to stimulate their germination and thereby blocking their mycelial growth by so doing preventing root infection from happening (Hawes et al., 2016). The interaction between root BCs and soil-borne microbes varies according to the plant species and the micro-organisms present. For example, root BCs from pea (*Pisum sativum*) are colonized by *Agrobacterium tumefaciens* whereas BCs from oat produce a mucilage which blocks and excludes bacteria from the BCs surface (Hawes et al., 2000). In addition, BCs were shown to synthesize and to secrete antimicrobial compounds (Driouich et al., 2013) such as phytoalexins, glycans/polymers and defense proteins that are able to fight against pathogens (Hawes et al., 2016). Arabinogalactan proteins (AGP) extracted from pea root cap and BCs prevent *in vitro* zoospores germination of *A. euteiches*. This could explain the reduced infection of the root cap that is observed compared to other RZs (Cannesan et al., 2012). Furthermore, the presence of extracellular DNA was reported within the mucilage surrounding the root BCs forming a Root Extracellular Trap (RET) by analogy with the Neutrophil Extracellular Trap (NET) in mammals (Driouich et al., 2013; Hawes et al., 2016; Tran T.M. et al., 2016). Additionally, it was demonstrated that BCs are also implicated in active induced defense strategies. Pathogens and/or elicitors cause an increase in the number of the BCs released into the rhizosphere as well as an enhanced secretion of metabolites by these cells (Curlango-Rivera et al., 2010; Cannesan et al., 2011). As a consequence of these studies, one can speculate that BCs activate defense mechanisms in greater intensity even before roots perceive MAMPS.

Interestingly, the plant model *A. thaliana* releases atypical BCs that do not separate individually but remain attached to each other and to the root cap. Due to this particular organization and mode of detachment, these cells are termed root border-like cells (BLCs). BLCs have recently been implicated in root defense but their functioning *in planta* is still unknown. However, Plancot et al., 2013 demonstrated that BLCs from *A. thaliana* are able to perceive MAMPs and activate defense responses by producing ROS as well as callose deposition. These findings suggest that BLCs are probably specifically involved in root defense in a similar manner to the root BCs.

## Stimulation of Root Defense

The growing need for more sustainable and environmentally safe agricultural practices requires the development of new agronomic approaches. In this context, the use of natural plant defense activators (or PDAs) has emerged as an effective and eco-friendly approach to agriculture (Benhamou and Rey, 2012; Delaunois et al., 2014). Upon infection, plants can also induce through molecular mechanisms systemic acquire resistance (SAR) in response to biotic and abiotic stimuli. This acquired resistance allows for a faster and stronger induction of defense pathways in response to subsequent pathogens. The plant is therefore “semi-permanently activated” for any further interactions with pathogenic organisms. PDAs are either small compounds or polymers that precondition the plant against further or other diseases and/or pathogen attack. When applied to plants, PDAs mimics pathogen aggression and trigger defense responses similar to those induce upon infection. The efficacy of PDAs relies on their ability to stimulate the plant defense systems mainly. They have little to no direct effect on pathogens themselves and so unlike pesticides avoid generating selective pressures on plant pathogen populations. Although, some PDAs such as chitosan can have some direct effects on certain pathogens (El Hassni et al., 2004) it seems in general this is not commonly observed for most PDAs. Overall PDAs represent good substitutes for conventional pesticides in food crops and have contributed to the growing research area of environmentally friendly “plant care” industries. Many commercially available compounds, when applied as foliar sprays act as inducers of plant defense and their efficiency has been demonstrated in field trial studies (Pichyangkura and Chadchawan, 2015; Malerba and Cerana, 2016). A list of such molecules used in field crops can be found in the review written by Thakur and Sohal (2013). These examples reflect the great interest in PDAs for controlling plant diseases using foliar applications whereas relatively few studies have focused on root treatments. Two main reasons could explain this discrepancy: (1) the complexity and difficulty of PDAs applications in soil and (2) the inability to control root bioavailability toward PDAs.

PDAs have been shown to induce strong and rapid defense responses when applied to roots. Several authors report the enhanced resistance to pathogens after root treatment with PDAs. This enhanced resistance in treated plants can be associated with various cellular and molecular defense responses (Badri et al., 2008; **Table 1**). Roots responded to several elicitors with ROS production characterized by a rapid release of H_2_O_2_ observed in the apoplast of various plant species (Zambounis et al., 2012). Activation of several enzyme activities implicated in ROS production (Nars et al., 2013), cell wall reinforcement (Mandal et al., 2009, 2013) and the production of antimicrobial compounds (Francis et al., 2009) have been reported following elicitor application (**Table 1**). In addition, different metabolic profiles are observed depending on the molecules being tested (Badri et al., 2008). These differences might be explained by cell specific transport mechanism that is induced in relation to the applied PDAs (Badri et al., 2008). For example, SA differentially regulates potassium, calcium, sulfate, ammonium as well as Major Facilitator Superfamily (MFS) and Multi-Antimicrobial Extrusion protein (MATE) transporters (Badri et al., 2008). Whereas MeJA was shown to differentially regulate MATE, an H^+^ ATPase pump, a sugar transporter and a metal transporter (Badri et al., 2008). Thus, root treatment with PDAs result in enhanced resistance to pathogens *via* the SAR mechanisms (Mandal et al., 2009). Furthermore, some molecules act differently as compared to elicitors, these are named priming agents. Activation of defense mechanisms under pathogen free-conditions is energetically taxing and competes with normal plant growth and development processes (Martinez-Medina et al., 2016). Priming does not induce the direct activation of defense mechanisms but activates defense signal pathways in plant cells without affecting plant growth (Aranega-Bou et al., 2014). For example, inactive mitogen-activated protein kinases (MAPKs) are typical defense signaling molecules that accumulate during priming. These molecules will be deployed when the plant is exposed to a pathogen or elicitor stress exposure (Beckers et al., 2009; Conrath, 2011) leading to a faster defense response. Beta-aminobutyric acid (BABA) is a well-known priming agent that potentiates (i.e., primes) SA-dependent defense responses in leaves and in roots (Zimmerli et al., 2000, **Table 1**). Elicitors including priming agents can induce both plant SAR. Such treatments can provide good substitute for pesticides in agriculture because they induce defense mechanisms in both treated and non-treated organs (Mandal et al., 2009; Myresiotis et al., 2014). Moreover, it was recently demonstrated that SAR is transmissible through the root system from SAR-triggered plants to their neighboring plants when treated with benzothiadiazole (BTH) (Song et al., 2010; Cheol Song et al., 2016). These results provide novel insights that can help in the development of new strategies for enhancing root defense capacities and also effectively inducing resistance against plant pathogens in target crops.

**Table 1:** Elicitors (including signaling compounds, priming agents and PDAs) that have been shown to induce plant defense after root treatment on different plant species.

Elicitors used	Species	Effects on roots	Application method	Reference
**PLANT DEFENSE ACTIVATORS**				
Acibenzolar-s-methyl(ASM)	*Citrus paradisi x Poncirus trifoliata*	- Reduced disease severity of citrus canker - PR-proteins production	Soil drench	Francis et al., 2009
	*Solanum lycopersicum*	- Reduction of nematode infestation	Root-dip or soil drench	Molinari and Baser, 2010
Benzothiadiazole (BTH)	*Gossypium hirsutum*	- ROS and PR-proteins production	Seedling immersion	Zambounis et al., 2012
	*Fragaria ananassa Duchense*	- Comparable *Podosphaera aphanis* disease control to foliar treatment	Soil drenching	Pertot et al., 2009
	*Carica papaya*	- Expression of defense-related enzymes - Reduction of *Phytophthora palmivora* symptoms - Increase of genes expression	Root drench	Zhu et al., 2003
Chitin oligosaccharide (CO)	*Arabidopsis thaliana*	- ROS production - Defense-related gene expression	Not indicated	Nars et al., 2013
Chitosan	*Lycopersicon esculentum*	- Cell wall reinforcement	Immersion of root fragment	Mandal and Mitra, 2007
	*Solanum melongena L.*	- Cell wall reinforcement - Increase of phenolic content	Immersion of root fragment	Mandal, 2010
	*Medicago truncalata*	- ROS production - Induction of defense-associated genes	Not indicated	Nars et al., 2013
	*Arabidopsis thaliana*	- ROS production - Defense-related gene expression	Not indicated	Nars et al., 2013
	*Solanum lycopersicum*	- Phenolic content increase - Reduced symptoms and disease incidence with *R. solanacearum* - Enzymes activity increase	Hydroponic culture	Mandal et al., 2013
	*Phoenix dactylifera L.*	- Increase of phenolic content - Changes in *Fusarium oxysporum* morphology - Increase of enzyme activity	Root injection	El Hassni et al., 2004
Saccharin	*Glycine max*	- Reduction of *P. pachyrhizi* disease severity	Root drench	Srivastava et al., 2011
**SIGNALING COMPOUNDS**				
Hexanoic acid	*Solanum lycopersicum*	- Protection against *Botrytis cinerea* - callose deposition in leaves	Hydroponic culture	Vicedo et al., 2009
Isonicotinic acid (INA)	*Citrus paradisi x Poncirus trifoliata*	- Reduced disease severity of citrus canker - PR-proteins production	Soil drench	Francis et al., 2009
Jasmonic acid (JA)	*Solanum lycopersicum*	- Phenolic content increase - Enzymes activity increase	Hydroponic culture	Mandal et al., 2013
	*Phoenix dactylifera L.*	- Enhanced *Fusarium oxysporum* resistance - Enzymes activity increase - ROS production	Root injection	Jaiti et al., 2009
	*Beta vulgaris L.*	- Synthesis of PR proteins, regulatory proteins, secondary metabolite biosynthetic enzymes, plant cell wall modifying proteins -Reduction of *Botrytis cinerea, Penicillium claviforme* and *Phoma betae* disease symptoms	Submerssion of roots	Fugate et al., 2012, 2017
Methyl salicylate (MeSA)	*Solanum melongena L.*	- Cell wall reinforcement - Increase of phenolic content	Immersion of root fragment	Mandal, 2010
	*Solanum lycopersicum*	- Reduction of nematode infestation	Root-dip or soil drench	Molinari and Baser, 2010
Methyl-Jasmonate (MeJA)	*Helianthus annuus L.*	- Increase in the activity of ROS implicated enzymes - ROS production (H_2_O_2_)	Seedling immersion	Parra-Lobato et al., 2009
	*Arabidopsis thaliana*	- Increase of phytochemical exudation - Gene expression modification	Seedling immersion	Badri et al., 2008
	*Solanum melongena L.*	- Cell wall reinforcement - Increase of phenolic content	Root fragment immersion	Mandal, 2010
	*Gossypium hirsutum*	- ROS production - PR-proteins production	Seedling immersion	Zambounis et al., 2012
	*Kalanchoe blossfeldiana*	- Anthocyanin accumulation	Roots were soaked in MeJA solution	Góraj-Koniarska et al., 2015
Nitric oxide (NO)	*Arabidopsis thaliana*	- Increase of phytochemical exudation - Gene expression modification	Seedling immersion	Badri et al., 2008
Salicylic acid (SA)	*Arabidopsis thaliana*	- Increase of phytochemical exudation - Gene expression modification	Seedling immersion	Badri et al., 2008
	*Solanum lycopersicum*	- Increase of the defense enzyme activity - Enhanced resistance against *Fusarium oxysporum* infection	Root feeding	Mandal et al., 2009
	*Solanum melongena L.*	- Cell wall reinforcement - Increase of phenolic content	Immersion of root fragment	Mandal, 2010
	*Solanum lycopersicum*	- Increase of SA content - Increase of enzymes activity - Reduce of disease symptoms	Hydroponic culture	Mandal et al., 2009
	*Solanum lycopersicum*	- Phenolic content increase - Reduced symptoms and disease incidence with *R. solanacearum* - Enzymes activity increase	Hydroponic culture	Mandal et al., 2013
	*Solanum lycopersicum*	- Protection against *Botrytis cinerea* - Callose deposition in leaves	Hydroponic culture	Vicedo et al., 2009
	*Solanum lycopersicum*	- Reduction of nematode infestation	Root-dip or soil drench	Molinari and Baser, 2010
	*Lycopersicon esculentum*	- Synthesis of PR-proteins - Induce PR-1 gene expression - Resistance against *A. solani*	Hydroponic vessels	Spletzer and Enyedi, 1999
**PRIMING AGENT**				
BABA	*Solanum lycopersicum*	- Protection against *Botrytis cinerea* - Callose deposition in leaves	Hydroponic culture	Vicedo et al., 2009
	*Arabidopsis thaliana*	- Enhanced resistance to *A. brassicicola* and *P. cucumerina* - Enhanced levels of resistance against *A. brassicicola* and *P. cucumerina* - Enhanced resistance to *P. cucumerina* and *A. brassicicola* - Callose deposition in leaves	Soil drench	Ton and Mauch-Mani, 2004

## Methods of Application and PDAs Efficacy

**FIGURE 2:**
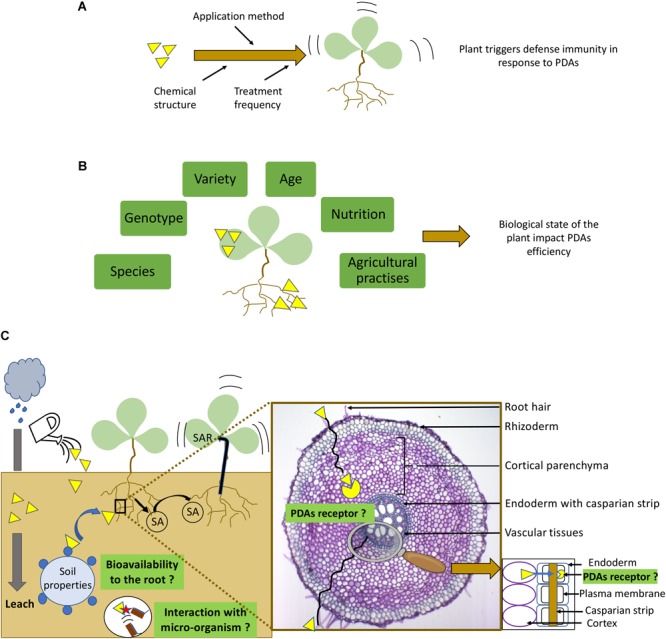
Overview of factors influencing plant defense stimulator (PDAs) efficiency in roots. **(A)** PDAs efficiency to trigger plant defense depends mainly on their chemical structure, on the application method and on treatment frequency. **(B)** Physiological state of the plant impact PDAs efficiency (Walters et al., 2013). PDAs effect can be different depending on species, plant genotype/variety, age of the plants and their vigor that depends on nutrition and agricultural practices. **(C)** In the environment, PDAs efficiency are dependent of physical and biological factors. For example, rain or irrigation can induce PDAs leaching. Soil properties and composition can improve PDAs bioavailability (e.g., humic acid) (Molinari and Loffredo, 2006; Molinari and Baser, 2010). Some microorganisms can degrade the PDAs *via* hydrolytic enzymes. When PDAs is absorbed by the plant, different root tissue can act as natural barrier reducing PDAs efficiency. As a consequence, receptor localization in the root is at the core of PDAs efficiency to trigger plant defense responses. However, it has to be noticed that once PDAs trigger plant responses, roots can release signal molecules such as salicylic acid (SA) (Cheol Song et al., 2016) to induce plant defense in neighboring plant and in non-treated organs such as leaves consisting in the systemic acquire resistance (SAR). 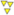: Plant defense activators 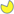: Plant defense activators receptors 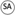: salicylic acid 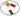: Interaction of plant defense activator with microorganisms

The use of PDAs is often controversial because of their variable efficiency as compared to pesticides which show a direct toxic action on the pathogenic organism. Although PDAs are effective in controlled laboratory conditions, efficacy in the field remains dependent on numerous factors (extrinsic and intrinsic). Variability can often be related directly to the specific PDAs used (**Figure 2A**). Chemical properties of the active compound are non-negligible parameters for efficacy. For example, biological activity of oligosaccharides like laminarin, a well-studied algal elicitor known to induce resistance, is highly dependent on their structures and degree of polymerization (Trouvelot et al., 2014). In addition, a chemical modification of sulphated laminarin, showed that the number of sulfate groups could influence its efficiency to induce plant defense responses (Menard, 2004, 2005; Gauthier et al., 2014). Indeed, the sulfate groups have been shown to protect the molecular core against enzymatic degradation (Menard, 2005). For an extensive review on the structure/activity relationships of carbohydrates see Trouvelot et al. (2014). It is also essential to consider the amount of product provided per plant, the time span for absorption and the application method used. The effect of the particular product used may be different if root uptake occurs from aqueous solutions or from the soil environment (Jakab et al., 2001). For example, induction of resistance to root-knot nematode in tomatoes was enhanced when SA was provided to plants as a soil drench rather than as root-dip (Molinari and Baser, 2010). Whereas the opposite occurred with Acibenzolar-*S*-methyl (ASM) treatments (Molinari and Baser, 2010; **Table 1**). The use of BABA for example as a soil drench is better tolerated by Arabidopsis without the deleterious effects of high concentrations used in foliar spray format (Jakab et al., 2001). The number of applications should also be considered in relation to the method of application (McGrann et al., 2017). When applied as a soil drench protective resistance lasts much longer as compared to a foliar spray (Francis et al., 2009). Similarly, when BION is applied as a soil drench it is a more efficient in its ability to reduce club-root development in brassica crops compared to applying it as a foliar spray (McGrann et al., 2017). PDAs efficiency (**Figure 2B**) is also dependent on the specific plant species being treated (Walters et al., 2013). In lettuce and cucumber, SA behaves as a phytotoxin by reducing root and shoot length (Pramanik et al., 2000) whereas in tomato, SA potentiates resistance (Molinari and Loffredo, 2006; Molinari and Baser, 2010). Environmental conditions can also affect plant vigor by reducing its capacity to trigger defense mechanisms and thereby impact PDAs bioavailabity. A case in point would be leaching of PDAs due to excessive rain (**Figure 2C**). Furthermore, soil properties and composition modulate the expression level of resistance of the plant and its response to PDAs treatment. For example, in tomato it was demonstrated that when SA was mixed with humic acid the induced resistance was more pronounced than when SA was provided alone (Molinari and Baser, 2010). The rhizosphere is highly enriched in microorganisms that can interact with PDAs in multiple ways. We can hypothesize that some of these microbes can degrade the PDAs *via* hydrolytic enzyme action resulting in a lower quantity that is bioavailable to the plant. However, to date, the role of microorganisms on the PDAs bioavailability remains unclear.

One of the most important challenges to the use of PDAs is their effective assimilation into the host (Paris et al., 2016). It should not be forgotten that the developmental stage of the treated organ, i.e., the root, is also an essential parameter to consider. With age, the root cortex becomes dry, corky and impermeable thus reducing PDAs absorption. To be recognized by the host, a PDAs must first be recognized by a receptor. As for Flg22, these receptors should be located within the inner layer of the endodermis. However, different root tissues can act as natural barriers reducing PDAs efficiency. Once absorbed by root, the molecule can migrate *via* the apoplastic or symplastic pathways. The apoplastic pathway involves cell wall transport. Therefore, the cell wall itself can block natural diffusion of PDAs into the cell and between associated compartments. For example, the exodermis is a thick and suberized barrier, which varies in degree depending on the species and age of the plant concerned. These properties might contribute to reducing the apoplastic transport of water and solutes. In contrast, molecules can migrate in the root using the symplastic pathway that allows for direct transport through the cytoplasm cells *via* the presence of plasmodesmata. Then, molecules can thus migrate from the rhizodermis cells through the cortex into the endodermis. Cortical parenchyma thickness thus have an impact on PDAs efficiency by dispersing molecules and lowering their final concentrations. The endodermis, which is another natural selective barrier, forces molecules to cross *via* symplastic flow due to the presence of casparian strip. The casparian strip is characterized by cell walls containing suberin and sometimes lignin. Due to its cell wall composition, the casparian strip regulates the apoplastic pathways acting as a filter which blocks solutes diffusion between the cortex and the vascular tissues. As a consequence, molecules must cross root endodermal cells. Therefore, the endodermis and the exodermis constitute tight barriers preventing the natural diffusion of molecules. Consequently, receptor localization in the root is at the core of PDAs efficiency in triggering plant defense responses. Therefore, we hypothesize here that these structures might participate in the variable and limited efficacy of PDAs root applications (**Figure 2C**).

## Concluding Remarks and Future Prospects

To date, root immunity is far from being fully understood and so many questions remain regarding mechanisms at the cellular and molecular levels. Interestingly, root defense response to elicitors and/or pathogen attacks exhibit tissue specificity. How such compartmentalization occurs in root defense as compared to leaves is still unclear. Is it due to distinct localization of signal receptors or only due to differences in the amplitude of responses in each tissue? One of the specificities of the root system is to release 100s and 1000s of living BCs in the surrounding environment. Due to their localization at the interface between root and soil, BCs are particularly important in root defense against pathogens. *A. thaliana* produces atypical BCs, so-called root border like-cells (BLCs) that remain attached to each other. Although the function of root BCs in root defense has been reported for different plant species, the exact function of root BLCs remains to be clearly determined. In *A. thaliana*, root BLCs were shown to perceive MAMPs and display defense mechanisms responses. These findings suggest that root BLCs are key elements in the root defense mechanism. Although BLCs production appear to be constitutively produced, no information is available regarding their formation and release upon biotic stress. Do they also produce specific defense molecules to form a root extracellular trap? Recent studies have confirmed that environmental signals can override the control of BCs production from pea roots. Such characteristic properties in BCs offers new and highly attractive prospects for the development of new defense strategies against pathogens. Thus, an exogenous treatment could increase the production of BCs and/or BLCs in order to cover and protect the elongation zone known to be the most sensitive to pathogens penetration. In this context, recent studies have demonstrated the potential of PDAs to trigger immunity in roots. Sometimes, such root treatments appear to be more efficient compared to foliar sprays. However, as compared to leaves, the efficiency of such elicitation in roots is dependent on multiple factors that influence the degree of induced resistance achieved, thus explaining why such treatment are still of limited use. Among these factors, soil properties associated with micro-organisms might undoubtedly impact PDAs bioavailability at the root level. Moreover, PDAs have to be recognized by receptors to trigger root defense. Although studies have reported strong activation of defense mechanisms after PDAs treatment, their receptor localization and mechanisms associated with the compartmentalized of defense in response to the elicitor remain unclear. Understanding the complex regulation of plant defense mechanisms and factors impacting PDAs efficiency is therefore of critical importance in order develop novel methods for disease prevention and prevention of disease spread.

## Author Contributions

The co-authors contribute to the writing of the review.

## Conflict of Interest Statement

The authors declare that the research was conducted in the absence of any commercial or financial relationships that could be construed as a potential conflict of interest.
